# *Drosophila melanogaster* behaviour changes in different social environments based on group size and density

**DOI:** 10.1038/s42003-020-1024-z

**Published:** 2020-06-12

**Authors:** Rebecca Rooke, Amara Rasool, Jonathan Schneider, Joel D. Levine

**Affiliations:** Department of Biology, University of Toronto at Mississauga, 3359 Mississauga Rd. North, Mississauga, ON L5L 1C6 Canada

**Keywords:** Behavioural genetics, Genotype

## Abstract

Many organisms, when alone, behave differently from when they are among a crowd. *Drosophila* similarly display social behaviour and collective behaviour dynamics within groups not seen in individuals. In flies, these emergent behaviours may be in response to the global size of the group or local nearest-neighbour density. Here we investigate i) which aspect of social life flies respond to: group size, density, or both and ii) whether behavioural changes within the group are dependent on olfactory support cells. Behavioural assays demonstrate that flies adjust their interactive behaviour to group size but otherwise compensate for density by achieving a standard rate of movement, suggesting that individuals are aware of the number of others within their group. We show that olfactory support cells are necessary for flies to behave normally in large groups. These findings shed insight into the subtle and complex life of *Drosophila* within a social setting.

## Abstract (web summary)

Rooke, Rasool et al. show that *Drosophila melanogaster* flies can detect the number of individuals around them and modify their behaviour based on group size and density. Furthermore, they show that LUSH-expressing cells are necessary for group size detection, suggesting the importance of olfactory support cells in modulating collective social behaviours.

## Introduction

In the wild, many animals interact and congregate into groups. It has been suggested that within a group, individuals follow simple rules based on local information and interact in ways that produce complex phenomena^1–4^. The benefits of forming groups can include increased foraging times^5,6^, lower predation risks^7–10^, social thermoregulation (e.g.^11,12^) and access to mates^13^. The composition of a group can influence the health and fitness of individuals within it^14–16^. Thus, understanding how the size and density of a group influence self-organisation can provide insight into the relative benefits of group living.

*Drosophila melanogaster* aggregate and form groups. *Drosophila* will aggregate even in the absence of food^17^. In addition, groups of flies display collective avoidance to aversive stimuli and increased foraging efficiency^18,19^. *Drosophila* groups disseminate social information throughout their life history, from larval communication and collective feeding^20,21^ to pheromonal communication during pupal metamorphosis^22^ to adult information transfer of oviposition preference^23^ and social learning^24^. Within this complex social aggregate, flies may regulate inter-individual distance^25^ and maintain a social space^26^, thereby limiting ʽrandomʼ encounters in favour of behavioural interactions^27^. Within aggregates, individual flies synchronise themselves to the group, both in terms of overall behaviour^28^ and pheromone profile^29^. Thus, flies adjust their behaviour and physiology to group size and composition.

There are at least two explanations for a fly's ability to sense and respond to the group: (1) with larger densities, the frequency of encounters between individuals increases locally and the fly adjusts to this frequency or (2) single flies are able to sense group size globally, such that their behaviour and physiology are influenced by the size of the group. These possibilities have been confounded in previous studies, which often do not separate the effects of group size and density. To disentangle their roles and gain insight into these key features of group dynamics and their underlying mechanisms, we investigate whether flies adjust their behaviour as a function of group size, density, or both using a social network approach. Moreover, we investigate whether olfaction may affect a group’s ability to adjust to different sizes and densities.

## Results

### Group size and density alter behaviour in wild-type flies

**Fig. 1:**
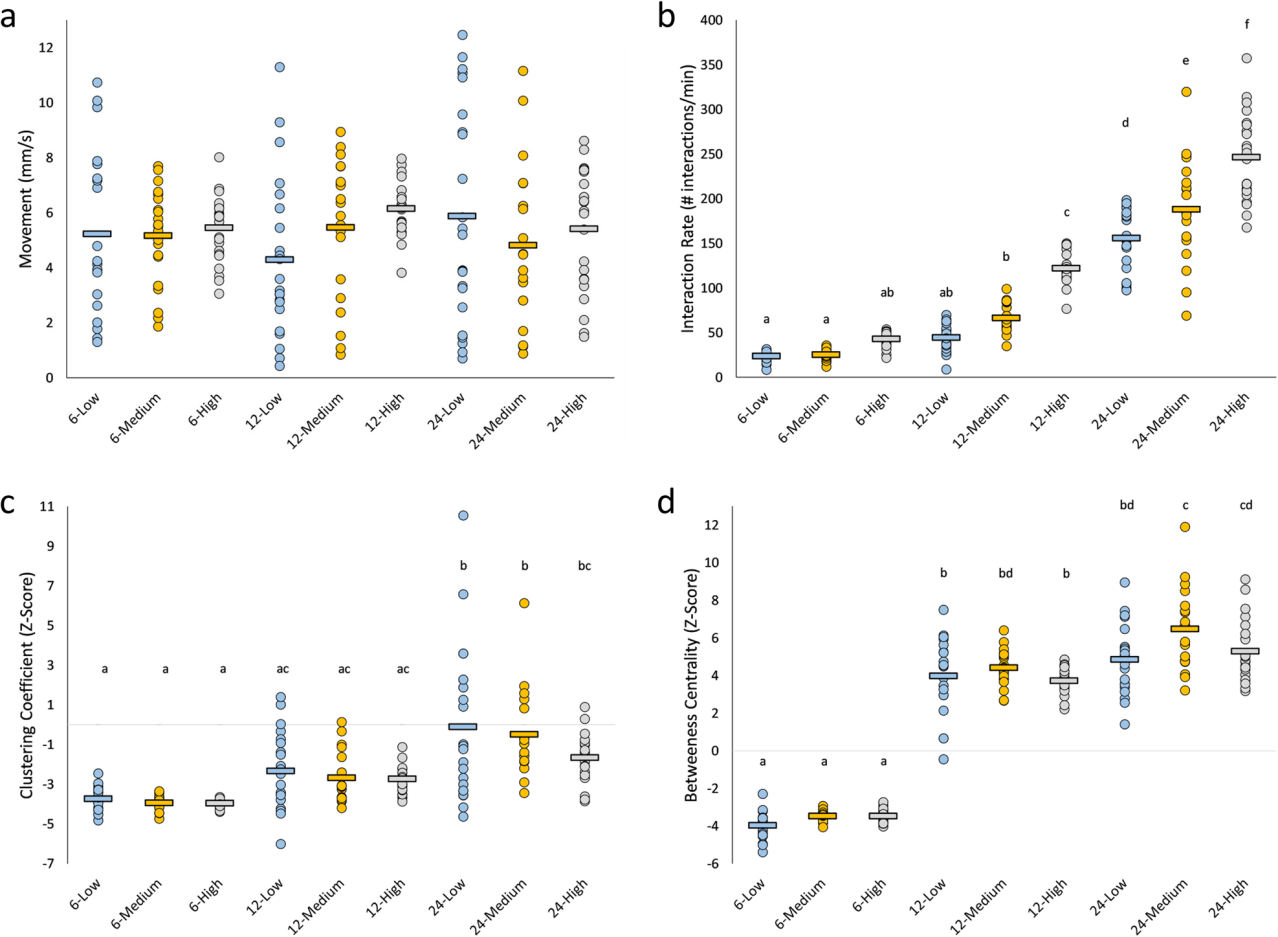
Behavioural properties of male flies at different densities and group sizes. Dots represent a single trial for groups of 6, 12 and 24 flies at low (blue), medium (orange) and high (grey) densities. The mean for each group size-density treatment is indicated by a horizontal line. Letters indicate statistical significance (α = 0.008) after outlier removal (Supplementary Fig. 3). **a** *Movement*. Movement does not change across different group sizes and densities. Density: *F*_(2,183)_ = 0.96, *p* = 0.386; Group size: *F*_(2,183)_ = 0.02, *p* = 0.982; Density × Group size: *F*_(4,183)_ = 1.52, *p* = 0.198. Group size 6: low—*n* = 20, medium—*n* = 21, high—*n* = 23; Group size 12: low—*n* = 22, medium—*n* = 20, high—*n* = 21; Group size 24: low—*n* = 23, medium—*n* = 19, high—*n* = 23. **b** *Interaction rate*. Interaction rates increase with increasing density and group size. Density: *F*_(2,184)_ = 78.27, *p* < 0.008; Group size: *F*_(2,184)_ = 538.27, *p* < 0.008; Density × Group size: *F*_(4,184)_ = 9.39, *p* < 0.008. Group size 6: low—*n* = 20, medium—*n* = 21, high—*n* = 24; Group size 12: low—*n* = 22, medium—*n* = 20, high—*n* = 22; Group size 24: low *n* = 22, medium = 19, high = 23. **c** *Clustering coefficient*. Flies in groups of 6 and 12 have lower clustering coefficient than groups of 24 flies. There is no effect of density on clustering coefficient. Density: *F*_(2,176)_ = 2.70, *p* = 0.070; Group size: *F*_(2,176)_ = 47.50, *p* < 0.008; Density × Group size: *F*_(4,176)_ = 1.10, *p* = 0.360. Group size 6: low *n* = 20, medium = 21, high = 21; Group size 12: low *n* = 21, medium = 20, high = 22; Group size 24: low *n* = 22, medium = 17, high = 21. **d** *Betweeness centrality*. Flies in groups of 6 have lower betweeness centrality than groups of 12 and 24 flies. Density: *F*_(2,178)_ = 6.03, *p* < 0.008; Group size: *F*_(2,178)_ = 809.95, *p* < 0.008; Density × Group size: *F*_(4,178)_ = 1.76, *p* = 0.140. Group size 6: low *n* = 20, medium = 21, high = 23; Group size 12: low *n* = 20, medium = 19, high = 22; Group size 24: low *n* = 21, medium = 18, high = 23.

First, we asked whether wild-type flies form social interaction networks (SINs) with different properties depending on the size and/or density of the group (Supplementary Fig. 1). Their interactions were characterised by use of an automated social interaction identification system^27^. For each group size, as density increases the interaction criteria (angle, distance, time) generally decreases, although due to the non-independent nature of how the criteria are calculated, no statistical tests can be done (see ref. ^27^). As group size increases while maintaining a constant density, the interaction criteria generally increases (Table 1; Supplementary Fig. 2). Thus, flies are adjusting the ways in which they interact based on the size and density of their groups. Next, movement and interaction rates were evaluated. Interestingly, flies across all group sizes and densities move the same amount (Fig. 1a). Intuitively, one would expect that increasing density would decrease individual movement via collision, with the increasing chance of physical encounters, whereas there would be little change in individual movement across constant densities. These results suggest that wild-type flies are regulating their movement to compensate for increasing densities. In addition, their rates of interaction are both group size- and density-dependent: as both group size and density increase, rates of interaction increase (Fig. 1b). Overall, these results indicate that flies are adjusting their interactions based on their group size and density, both in terms of the rate at which they interact and the way they interact, while regulating their movement to compensate for density.

**Table 1:**
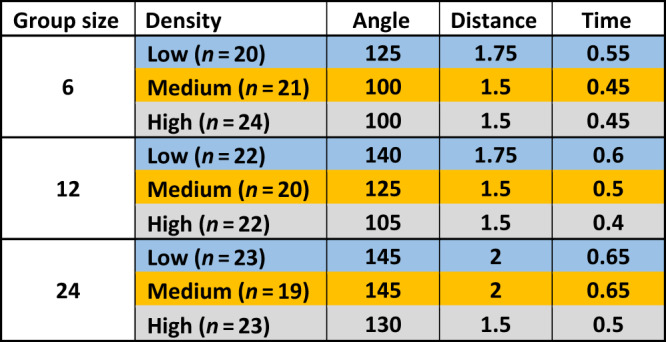
Interaction criteria for wild-type flies for different group sizes and densities.

The median angle and distance at which wild-type flies interact and the median duration of an interaction were determined by an automated system ^27^. We calculated interaction criteria for groups of 6, 12 and 24 flies at low (blue), medium (orange) and high (grey) densities.

Next, two SIN properties were evaluated: (1) *clustering coefficient*: a measure of how interconnected neighbours are to one another^30^ and (2) *betweeness centrality*: a measure of network cohesion^30^. For clustering coefficient, groups of six and 12 behave similarly to each other and have a lower clustering coefficient than groups of 24 flies (Fig. 1c). The higher clustering coefficient in groups of 24 indicates that flies are more interconnected when in a large group. For betweeness centrality, groups of six flies form SINs with lower betweeness centrality than groups of 12 and 24 flies (Fig. 1d). This indicates that when flies are in medium- and large-sized groups, they form SINs with greater network cohesion. Overall, these data indicate that flies can evaluate their social environment and alter their behaviour to regulate for differences in density and group size, suggesting that flies respond to their group using both a local and global approach.

### LUSH-mediated olfaction regulates behaviour across groups

**Fig. 2:**
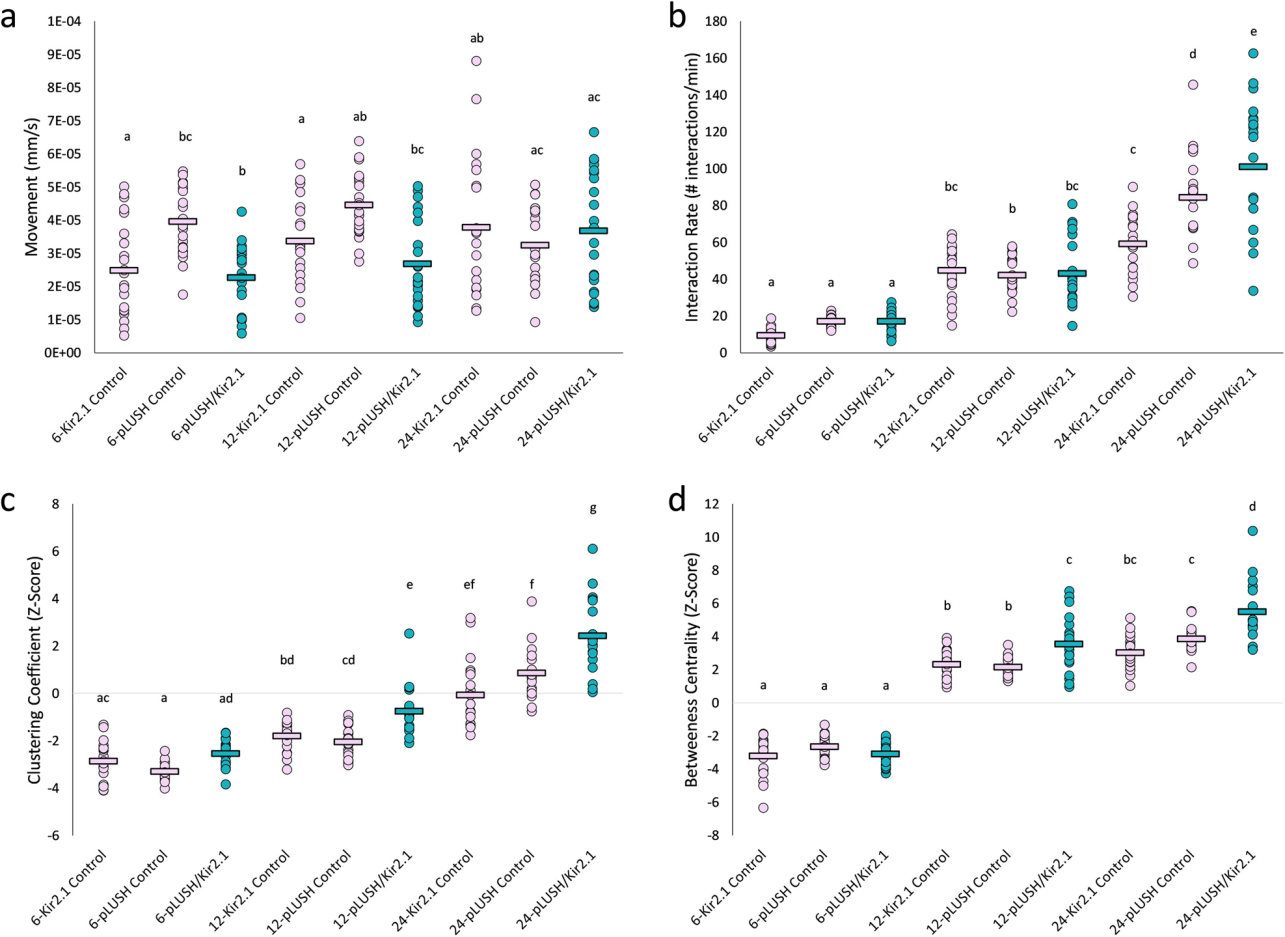
Behavioural properties of male flies with inhibited *lush*-expressing cells at different group sizes. Dots represent a single trial for groups of 6, 12 and 24 flies for UAS-Kir2.1 control flies (pink), *lush*-GAL4 control flies (pink) and silenced *lush* flies (turquoise) at medium density. The mean for each group size-genotype is indicated by a horizontal line. Letters indicate statistical significance (α = 0.008) after outlier removal (Supplementary Fig. 4). **a** *Movement*. The movement for the silenced *lush* flies (turquoise) is the same as at least one of their respective controls (pink) for each group size. Genotype: *F*_(2,184)_ = 6.15, *p* < 0.008; Group size: *F*_(2,184)_ = 4.61, *p* = 0.011; Genotype × Group size: *F*_(4,184)_ = 6.93, *p* < 0.008. Group size 6: UAS-Kir2.1 Control—*n* = 22, Gal4 Control—*n* = 22, Experimental—*n* = 22; Group size 12: UAS-Kir2.1 Control—*n* = 22, Gal4 Control—*n* = 21, Experimental—*n* = 22; Group size 12-UAS-Kir2.1 Control *n* = 21, Gal4 Control = 19, Experimental = 22. **b** *Interaction rate*. The interaction rates for the silenced *lush* flies (turquoise) are the same as at least one of their respective controls (pink) for each group size. Genotype: *F*_(2,183)_ = 13.39, *p* < 0.008; Group size: *F*_(2,183)_ = 229.88, *p* < 0.008; Genotype × Group size: *F*_(4,183)_ = 9.2, *p* < 0.008. Group size 6: UAS-Kir2.1 Control—*n* = 22, Gal4 Control—*n* = 22, Experimental—*n* = 22; Group size 12: UAS-Kir2.1 Control—*n* = 22, Gal4 Control—*n* = 21, Experimental—*n* = 22; Group size 24: UAS-Kir2.1 Control *n* = 20, Gal4 Control = 19, Experimental = 22. **c** *Clustering coefficient*. Silenced *lush* flies (turquoise) in groups of 6 do not differ in clustering coefficient from their controls (pink). When in groups of 12 and 24, silenced *lush* flies have higher clustering coefficient than their controls. Genotype: *F*_(2,167)_ = 30.68, *p* < 0.008; Group size: *F*_(2,167)_ = 243.49, *p* < 0.008; Genotype × Group size: *F*_(4,167)_ = 6.63, *p* < 0.008. Group size 6: UAS-Kir2.1 Control—*n* = 20, Gal4 Control—*n* = 20, Experimental—*n* = 21; Group size 12: UAS-Kir2.1 Control—*n* = 19, Gal4 Control—*n* = 21, Experimental—*n* = 18; Group size 24: UAS-Kir2.1 Control—*n* = 20, Gal4 Control—*n* = 18, Experimental—*n* = 19. **d** *Betweeness centrality*. Silenced *lush* flies (turquoise) in groups of 6 do not differ in betweeness centrality from their controls (pink). When in groups of 12 and 24, silenced *lush* flies have higher betweeness centrality than their controls. Genotype: *F*_(2,172)_ = 18.52, *p* < 0.008; Group size: *F*_(2,172)_ = 710.79, *p* < 0.008; Genotype × Group size: *F*_(4,172)_ = 9.28, *p* value < 0.008. Group size 6: UAS-Kir2.1 Control—*n* = 22, Gal4 Control—*n* = 20, Experimental—*n* = 21; Group size 12: UAS-Kir2.1 Control—*n* = 21, Gal4 Control—*n* = 21, Experimental—*n* = 20; Group size 24: UAS-Kir2.1 Control—*n* = 19, Gal4 Control—*n* = 18, Experimental—*n* = 19.

We asked whether olfaction is required for flies to evaluate the number of individuals in their group, while maintaining a fixed density. To do this, cells expressing the olfactory binding protein, LUSH, were inhibited. LUSH is expressed in all *Drosophila* trichoid sensillae^31^ and facilitates the binding of ligands to olfactory receptors^31,32^. For all measurements, control flies behave similarly to wild-type flies, suggesting that we are capturing a robust group-size effect (Figs. 1 and 2). In *lush*-inhibited flies, interaction criteria exhibit a similar trend to those of wild-type flies: at constant density, the interaction criteria generally increase with group size (Table 2). Although movement is genotype-dependent, there was no group-size effect on movement: the experimental *lush*-inhibited flies move the same amount as at least one of their respective control lines (Fig. 2a). We see a similar trend of interaction rates when compared with wild-type flies, where increasing group size increases the interaction rates (Figs. 1b and 2b). In groups of six, inhibiting *lush*-expressing cells has no effect on clustering coefficient or betweeness centrality. However, when *lush*-expressing cells are inhibited in flies in groups of 12 or 24, flies organise themselves in ways that mimic a larger group (Fig. 2c,d). Interestingly, for both clustering coefficient and betweeness centrality, *lush*-inhibited flies in groups of 12 have a higher clustering coefficient and betweeness centrality than their controls and behave as if they are in a group of 24. Groups of 24 *lush*-inhibited flies have higher clustering coefficient and betweeness centrality values than their controls, presumably behaving like they are in an even larger group (Fig. 2c,d). Overall, we observe that, in medium- and large-sized groups, olfaction is required for flies to accurately detect the number of individuals in their social environment and, when inhibited, flies behave as if in a larger group. Moreover, impairing olfaction has no effect on flies in small groups, indicating that other sensory modalities may be used to evaluate small group sizes.

**Table 2:**
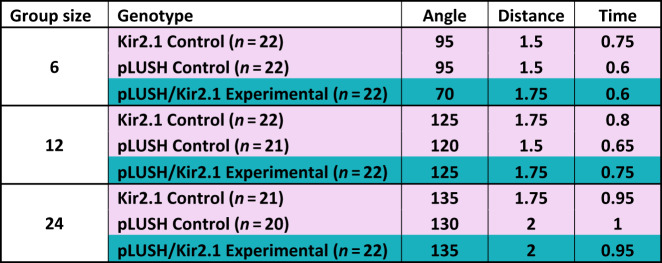
Interaction criteria for *lush*-silenced flies and their controls for different group sizes at medium density.

The median angle and distance at which flies interact and the median duration of an interaction were determined by an automated system^27^. We calculated interaction criteria for groups of 6, 12 and 24 flies at medium density for Kir2.1 UAS control flies (pink), pLUSH-Gal4 control flies (pink) and *lush*-silenced Kir2.1/pLUSH experimental flies (turquoise).

## Discussion

In this paper, we show that flies can detect the number of individuals around them and, importantly, their behaviour changes depending on that number. We test group size and density separately and show that some, but not all, group behaviours are density-dependent. A fly's ability to sense and respond to the group is not merely a function of flies encountering each other more frequently: although their interaction rates and interaction criteria change based on both group size and density, their social network properties were primarily dependent on group size. Thus, individual group members can sense their social environment and are influenced by the size of their group. Moreover, we show that *lush*-expressing cells are necessary for detecting group size. LUSH is required to detect cis-vaccenyl acetate (cVA), a male-specific volatile pheromone, which is known to cause aggregation and dispersal in *Drosophila*^33,34^. It is possible that cVA is a necessary component for flies to accurately assess group size but that it is not the only factor necessary: when *lush*-expressing cells are inhibited, flies in groups of six show no effect in their SIN properties. This indicates that flies in small groups may rely on non-olfactory mechanisms for sensing the social environment, such as vision, mechanosensation or auditory cues. Previous studies on groups of 12 flies failed to show effects of vision or sound on group-level structure^35^. However, recent research indicates that both vision and the cVA receptor, Or65a, are required to regulate social group interactions in groups of 10 flies^36^ and groups of 50 flies’ social clustering is impacted by various sensory modalities, including olfaction, vision and touch^17^. Further studies on smaller group sizes are required to fully dissect how flies detect their social environment.

Individuals following simple rules may yield complex group responses despite the absence of a centralised or global control mechanism, a phenomenon defined as self-assembly^37^. At the group level, this can manifest as insect swarms, fish shoals or avian flocks^1–3,38^. The group size-dependent behaviour that we observe for betweeness centrality is seen in shoaling fish, where group sizes greater than six have different foraging properties in the presence of a predator compared with groups of six or less^39^. Similar to our finding of movement across different densities, ants have also been shown to regulate their behaviour based on density^40^. Recent research shows that SINs are formed by various species of *Drosophila* and their network properties differ across species^41^. Thus, social regulation at the group-level spans across species, taxa and across a variety of social systems. Perhaps because flies are small and move at a quick time scale, most research has not focused on what happens within a group of flies. Yet, it is clear that *Drosophila melanogaster* has an innate capacity to assess its social environment and participate in complex group behaviour.

## Methods

### Fly stocks

The Canton-S strain was the wild-type strain of *D. melanogaster*. For the *lush* experiments, we backcrossed both our *lush*-GAL4 line (w*;pLUSH-GAL4;sb/Tm6B) and UAS-Kir2.1 line (BDSC: 6596 w*;P{UAS-Hsap\KCNJ2.EGFP}1;+) to *w*^*1118*^. We used *w*^*1118*^*;pLUSH-GAL4/+;+/+* male progeny as Gal4 control flies. We used *w*^*1118*^*;P{UAS-Hsap\KCNJ2.EGFP}1/+;+/+* as UAS control flies. The genotype of our experimental flies was *w*^*1118*^*; pLUSH-Gal4/ P{UAS-Hsap\KCNJ2.EGFP}1;+/+*.

### Video acquisition and fly treatment

Experiments were acquired as described in Schneider et al.^35^. Briefly, fly stocks were maintained at 25 °C in a 12/12 Light–Dark cycle. Collections were done under light anesthesia (CO_2_) within 4 h of eclosion. The appropriate numbers of male flies were housed for 3 days at 25 °C in a 12/12 Light–Dark cycle in vials with food. Flies were housed in group sizes determined by treatment. Experiments were performed within a 2-h window starting 3 h before lights off. Flies were gently aspirated by mouth into plexiglass arenas and were allowed to acclimate for 10 min prior to the 30-min video acquisition. Video acquisition was performed in an environmental chamber (25 °C, 60% humidity) using Fview software (open source, Ubuntu package used) and FireflyMV (Point Grey) cameras. Each assay was performed on a distinct sample of flies. Flies were discarded after the 30-min video was recorded.

Arena diameters are described in Supplementary Fig. 1. The pLUSH-Kir2.1 experiment was performed at medium density for all group sizes.

### Social interaction networks (SINs)

These experiments were performed as described by Schneider et al.^35^. Briefly, each flies’ trajectory was tracked from the videos using Ctrax (open source, versions 0.3.2 and 0.5.18) to track fly movement, orientation and identity. Ctrax-Fixerrors was used to manually inspect/correct fly trajectories. Custom algorithms were used to generate the SINs at 25% network density. Each network iteration had its structural measure calculated, and then normalised against 10,000 random networks which preserved the in- and out- degree of the iteration to create a *Z*-score.

Social distance and social interaction space were determined using an open-source automated algorithm described by Schneider & Levine^27^ in Matlab (Mathworks).

To generate ‘null’ datasets, we mixed and matched trajectories within a treatment to generate virtual trials which contained the appropriate number of flies. These trials, therefore, controlled for the movement and behaviour of flies in our arena without social feedback. The ‘null’ dataset’s connectivity matrices and measurements were calculated as above.

To control for the artificial change in network measures caused by varying the network size, the *Z*-scores of our observed networks were normalised a second time against the average mean and average standard deviation of the respective ‘null’ measurements.

Each trial (or *n* = 1) indicates the mean from an independent group of flies (at their correct group size/density/genotype) that were discarded after the 30-min network experiment was acquired. For each experimental treatment, we acquired videos from ~20 independent groups of flies (*n* = ~20 for each treatment).

### Statistics and reproducibility

We analyzed four network properties, movement and interaction rate. For each experiment, a two-way ANOVA was performed in Matlab (MathWorks) with a Bonferroni corrected alpha (α = 0.008) followed by a Tukey–Kramer post hoc (α = 0.05). Each data point indicates a mean from a single trial, derived from a group of flies that were filmed once and then disposed of. For all data, outliers ≥75th quartile+(1.5 × IQR) or ≤25th quartile-(1.5 × IQR) were removed (Supplementary Figs. 3 and 4) before statistical testing.

Sample sizes for the wild-type experiment before outlier removal are as follows: Group size 6: low—*n* = 20, medium—*n* = 21, high—*n* = 24; Group size 12: low—*n* = 22, medium—*n* = 20, high—*n* = 22; Group size 24: low—*n* = 23, medium—*n* = 19, high—*n* = 23.

Sample sizes for the LUSH experiment before outlier removal are as follows: Group size 6: UAS-Kir2.1 Control—*n* = 22, Gal4 Control—*n* = 22, Experimental—*n* = 22; Group size 12: UAS-Kir2.1 Control—*n* = 22, Gal4 Control—*n* = 21, Experimental—*n* = 22; Group size 24: UAS-Kir2.1 Control—*n* = 21, Gal4 Control—*n* = 20, Experimental—*n* = 22.

### Reporting summary

Further information on research design is available in the Nature Research Reporting Summary linked to this article.

## Supplementary information

Supplementary Information

Supplementary Data

Reporting Summary

Description of Additional Supplementary Files

## Data Availability

The datasets generated during and/or analyzed during the current study are provided as Supplementary Data.

## Code availability

Custom code used in this manuscript is available upon request from the corresponding author.
