# Supplementary material for: Drosophila melanogaster behaviour changes in different social environments based on group size and density
Source: Commun Biol. 2020 Jun 12;3:304. doi: 10.1038/s42003-020-1024-z (PMC7293324; doi:10.1038/s42003-020-1024-z)
Supplement: Supplementary file 1 — Supplementary Information [file 42003_2020_1024_MOESM1_ESM.pdf]

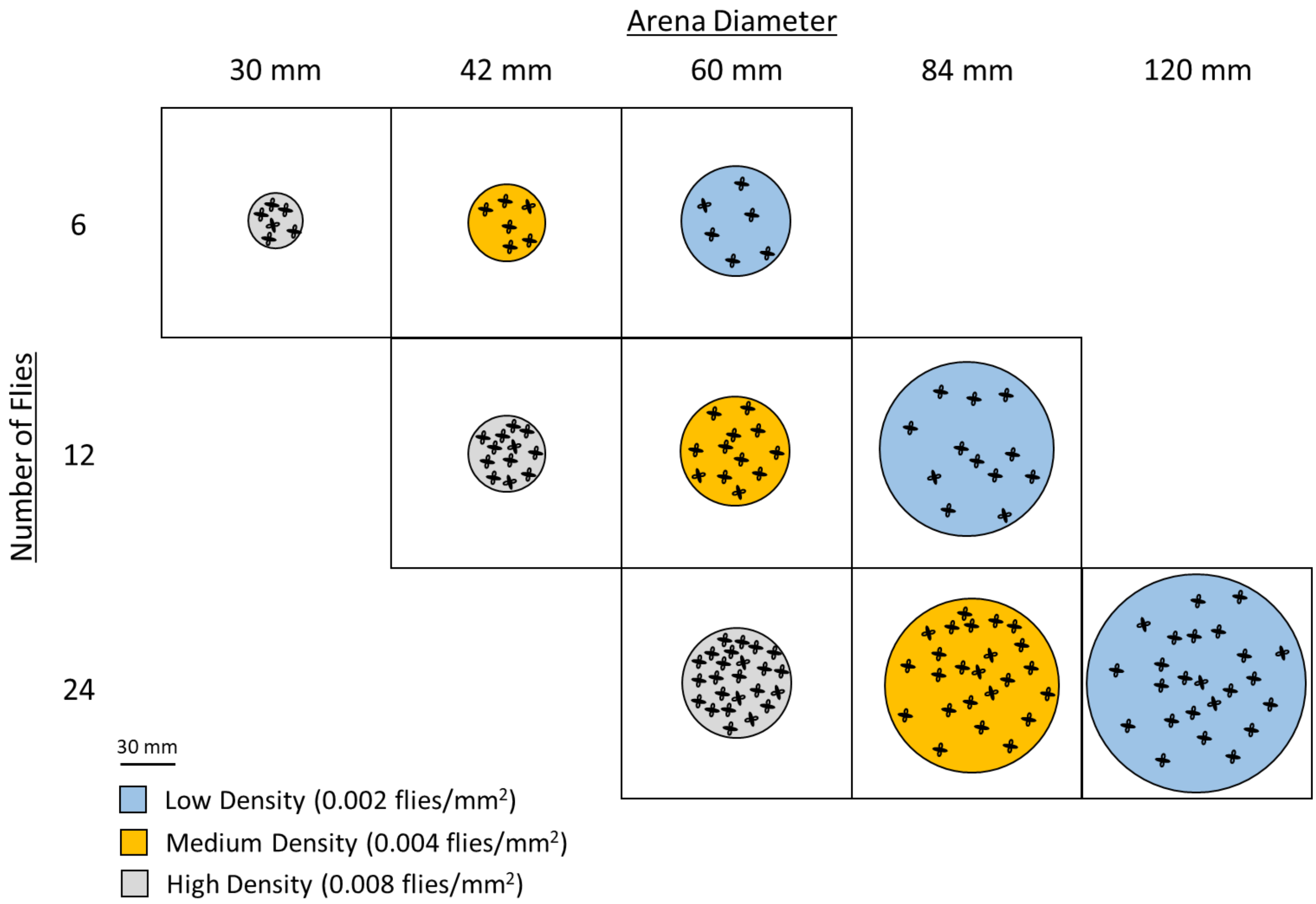

Supplementary Figure 1: Depiction of the arenas and flies used in this experiment. Each arena's diameter is listed above in millimeters, while the number of wildtype flies is listed on the left. Each density is depicted by the colors grey (high density), orange (medium density), and blue (low density).

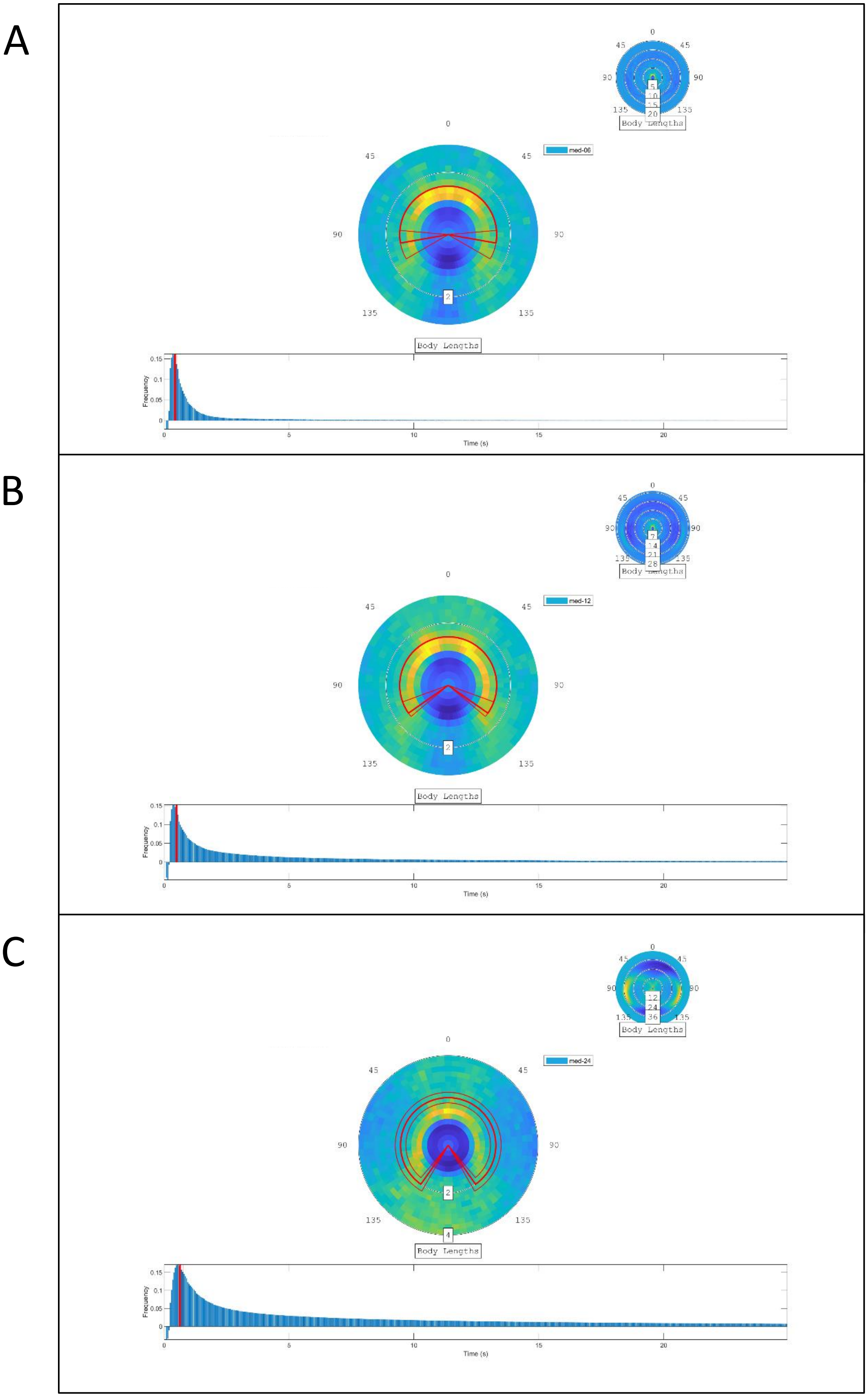

A
med-06
Body Lengths
Frequency
Time (s)
B
med-12
Body Lengths
Frequency
Time (s)
C
med-24
Body Lengths
Frequency
Time (s)

Supplementary Figure 2: Estimated distance, angle and time criteria of wildtype male flies at medium density and various group sizes. Circular heat maps represent the distribution of spatial positions of flies relative to a single fly in the center. Numbers inside the circle represent distance positions (in body lengths) and numbers outside the circle represent angle positions (in degrees). Red regions represent social interaction hot spots while blue regions represent social interaction cold spots. The angle and distance bins that capture the over-representation in social positioning is indicated by the bolded red outline, which we call the social space. The two non-bolded outlines are the lower and upper limits of the 95% confidence interval obtained through bootstrapping social space estimates. The histograms below each heat map represent the distribution of the time flies spend interacting using the distance and angle criteria extrapolated above. The bolded red line indicates the time estimate and additional lines represent the 95% confidence interval obtained through bootstrapping. **A) Groups of six wildtype male flies**. Flies were filmed at medium density. n=21 **B) Groups of twelve wildtype male flies.** Flies were filmed at medium density. n=20 **C) Groups of twenty-four wildtype male flies.** Flies were filmed at medium density. n=19. All figures were generated using an automated method from Schneider & Levine (2014).

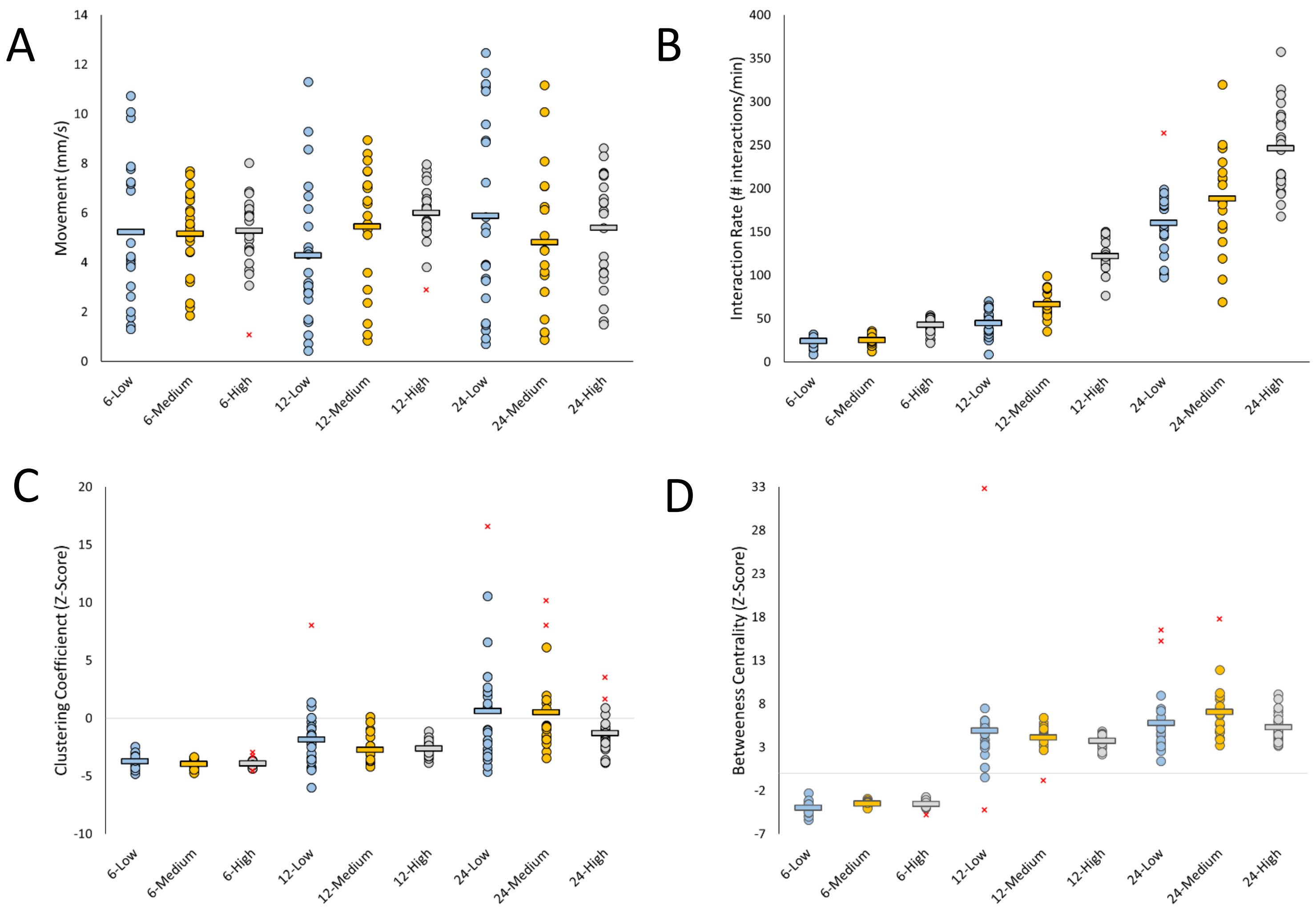

Supplementary Figure 3: Behavioural properties of male flies at different densities and group sizes (with outliers). Means (of all data, including outliers) of each property for groups of six, twelve and twenty-four flies at low (blue), medium (orange) and high (gray) densities are indicated by straight lines. Outliers are indicated by red "X". Data was considered an outlier if it was ≥75th quartile+(1.5*IQR) or ≤25th quartile-(1.5*IQR). **A) Movement** The amount of movement (mm/s) exhibited by flies belonging to their respective group size and density. **B) Interaction Rate** The rate of interactions (# interactions/min) exhibited by flies belonging to their respective group size and density. **C) Clustering Coefficient** The clustering coefficient (z-scores), a measure of how interconnected neighbours are to one another, for each group size and density treatment. **D) Betweeness Centrality** The betweeness centrality (z-scores), a measure of network cohesion, for each group size and density treatment. Sample sizes before outlier removal are as follows: Group Size Six: Low-n=20, Medium-n=21, High-n=24; Group Size Twelve: Low-n=22, Medium-n=20, High-n=22; Group Size Twenty-Four: Low-n=23, Medium-n=19, High-n=23.

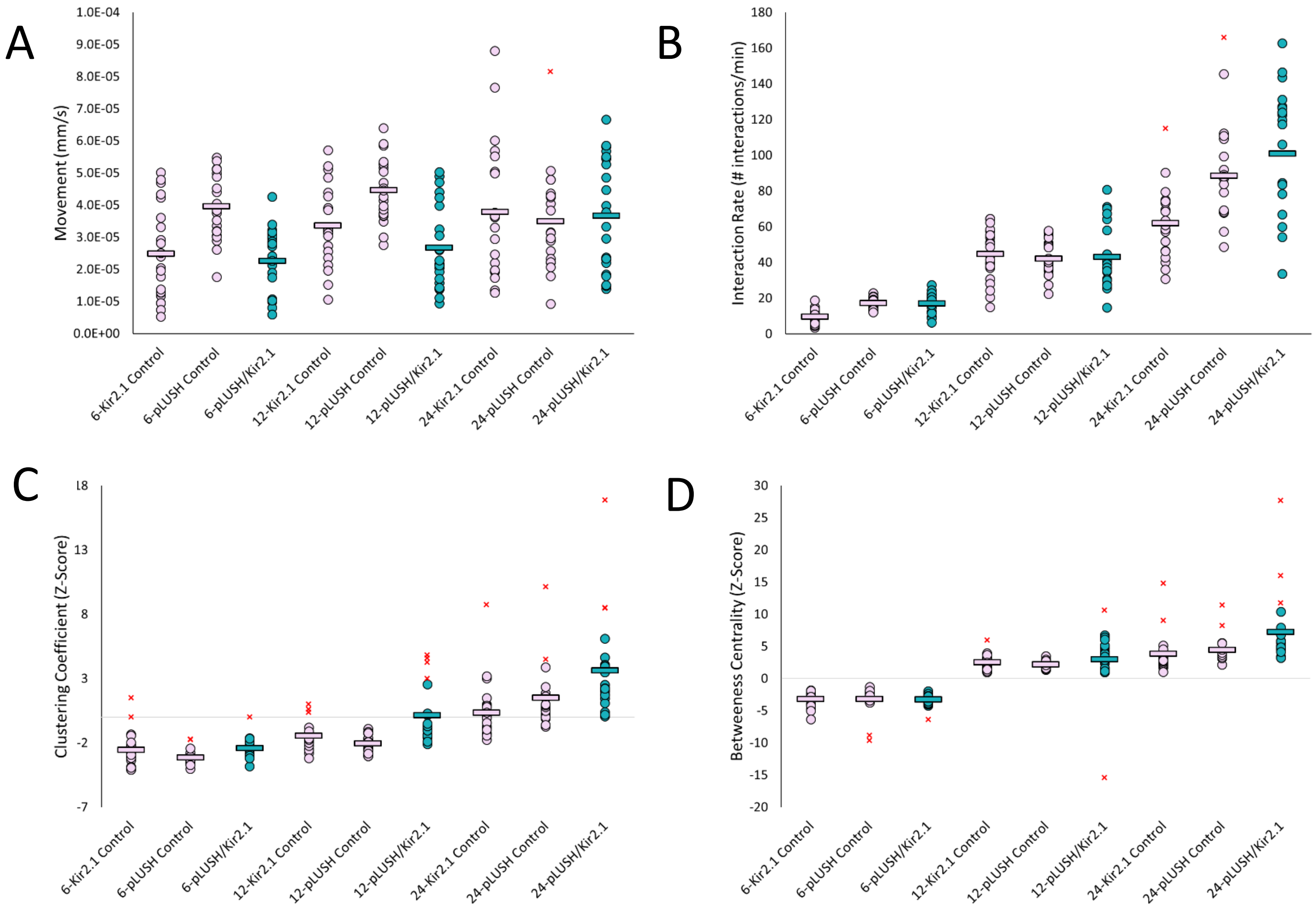

Supplementary Figure 4: Behavioural properties of male flies with inhibited *lush*-expressing cells at different group sizes (with outliers). Means (of all data, including outliers) of each property for groups of six, twelve and twenty-four flies for UAS Kir2.1 control flies (pink), *lush*-GAL4 control flies (pink) and silenced *lush* flies (turquoise) at medium density are indicated by straight lines. Outliers are indicated by red "X". Data was considered an outlier if it was ≥75th quartile+(1.5*IQR) or ≤25th quartile-(1.5*IQR). **A) Movement** The amount of movement (mm/s) exhibited by control and experimental flies belonging to their respective group size. **B) Interaction Rate** The rate of interactions (# interactions/min) exhibited by control and experimental flies belonging to their respective group size. **C) Clustering Coefficient** The clustering coefficient (z-scores), a measure of how interconnected neighbours are to one another, for control and experimental flies belonging to each group size. **D) Betweeness Centrality** The betweeness centrality (z-scores), a measure of network cohesion, for control and experimental flies belonging to each group size. No statistical tests were performed. Sample sizes before outlier removal are as follows: Group Size Six: UAS-Kir2.1 Control-n=22, Gal4 Control-n=22, Experimental-n=22; Group Size Twelve: UAS-Kir2.1 Control-n=22, Gal4 Control-n=21, Experimental-n=22; Group Size Twenty-Four: UAS-Kir2.1 Control-n=21, Gal4 Control-n=20, Experimental-n=22.
