# Supplementary material for: Drosophila melanogaster behaviour changes in different social environments based on group size and density
Source: Commun Biol. 2020 Jun 12;3:304. doi: 10.1038/s42003-020-1024-z (PMC7293324; doi:10.1038/s42003-020-1024-z)
Supplement: Supplementary file 3 — Reporting Summary [file 42003_2020_1024_MOESM3_ESM.pdf]

nature research

Corresponding author(s): Joel D. Levine

Last updated by author(s): May 16, 2020

Reporting Summary

Nature Research wishes to improve the reproducibility of the work that we publish. This form provides structure for consistency and transparency in reporting. For further information on Nature Research policies, see Authors & Referees and the Editorial Policy Checklist.

Statistics

For all statistical analyses, confirm that the following items are present in the figure legend, table legend, main text, or Methods section.

n/a	Confirmed	
☐	☒	The exact sample size (*n*) for each experimental group/condition, given as a discrete number and unit of measurement
☐	☒	A statement on whether measurements were taken from distinct samples or whether the same sample was measured repeatedly
☐	☒	The statistical test(s) used AND whether they are one- or two-sided<br>*Only common tests should be described solely by name; describe more complex techniques in the Methods section.*
☐	☒	A description of all covariates tested
☐	☒	A description of any assumptions or corrections, such as tests of normality and adjustment for multiple comparisons
☐	☒	A full description of the statistical parameters including central tendency (e.g. means) or other basic estimates (e.g. regression coefficient) AND variation (e.g. standard deviation) or associated estimates of uncertainty (e.g. confidence intervals)
☐	☒	For null hypothesis testing, the test statistic (e.g. *F*, *t*, *r*) with confidence intervals, effect sizes, degrees of freedom and *P* value noted<br>*Give P values as exact values whenever suitable.*
☒	☐	For Bayesian analysis, information on the choice of priors and Markov chain Monte Carlo settings
☒	☐	For hierarchical and complex designs, identification of the appropriate level for tests and full reporting of outcomes
☒	☐	Estimates of effect sizes (e.g. Cohen's *d*, Pearson's *r*), indicating how they were calculated

Our web collection on statistics for biologists contains articles on many of the points above.

Software and code

Policy information about availability of computer code

Data collection	Fview software (open source, Ubuntu package used) was used to record the videos of the flies.
Data analysis	Ctrax (open source, versions 0.3.2 and 0.5.18) was used to track fly movement, orientation and identity. Ctrax-Fixerrors was used to manually inspect/correct fly trajectories. An automated algorithm [open source; Schneider & Levine (2014), Biol. Lett.] was used to determine the social spacing parameters for each group and genotype. Custom algorithms were used to generate the Social Iterative Networks (SINs).

For manuscripts utilizing custom algorithms or software that are central to the research but not yet described in published literature, software must be made available to editors/reviewers. We strongly encourage code deposition in a community repository (e.g. GitHub). See the Nature Research guidelines for submitting code & software for further information.

Data

Policy information about availability of data

All manuscripts must include a data availability statement. This statement should provide the following information, where applicable:

- Accession codes, unique identifiers, or web links for publicly available datasets
- A list of figures that have associated raw data
- A description of any restrictions on data availability

The datasets generated during and/or analysed during the current study are available from the corresponding author on reasonable request.

Field-specific reporting

Please select the one below that is the best fit for your research. If you are not sure, read the appropriate sections before making your selection.

☐ Life sciences ☒ Behavioural & social sciences ☐ Ecological, evolutionary & environmental sciences

For a reference copy of the document with all sections, see nature.com/documents/nr-reporting-summary-flat.pdf

Behavioural & social sciences study design

All studies must disclose on these points even when the disclosure is negative.

Study description	Video data was acquired from Drosophila. Their movement was video recorded for 30 minutes and tracked using Ctrax (open source, versions 0.3.2 and 0.5.18) software. An automated algorithm [open source; Schneider & Levine (2014), Biol. Lett.] was used to determine the social spacing parameters for each group and genotype. Custom algorithms were used to generate the Social Iterative Networks (SINs). Statistics were performed in Matlab. Approximately 20 independent replicates were used for each group.
Research sample	Male wild-type Canton-S Drosophila were used in the first experiment. For the lush experiment, we used males from 3 genotypes (described in methods): (A) w1118;pLUSH-GAL4/+;+/+ (B) w1118;P{UAS-Hsap\KCNJ2.EGFP}1/+;+/+ (C) w1118;pLUSH-GAL4/P{UAS-Hsap \KCNJ2.EGFP}1;+/+ All males were collected shortly after eclosion and stored at 25C in a 12h L/D cycle for 3 days before being used for experiments.
Sampling strategy	We used an n~=20 based on empirical results.
Data collection	Videos were recorded using FireflyMV (Point Gray) firewire cameras using fview software. Cameras were backlit using IR light.
Timing	April 15th 2019-May 17th 2019
Data exclusions	Outliers were removed from all data sets. Data was considered an outlier if it >= 75th quartile+(1.5xIQR) or <=25th quartile-(1.5xIQR)
Non-participation	N/A
Randomization	For each experiment, flies were randomly chosen (according to genotype).

Reporting for specific materials, systems and methods

We require information from authors about some types of materials, experimental systems and methods used in many studies. Here, indicate whether each material, system or method listed is relevant to your study. If you are not sure if a list item applies to your research, read the appropriate section before selecting a response.

Materials & experimental systems

n/a	Involved in the study	
☒	☐	Antibodies
☒	☐	Eukaryotic cell lines
☒	☐	Palaeontology
☐	☒	Animals and other organisms
☒	☐	Human research participants
☒	☐	Clinical data

Methods

n/a	Involved in the study	
☒	☐	ChIP-seq
☒	☐	Flow cytometry
☒	☐	MRI-based neuroimaging

Animals and other organisms

Policy information about studies involving animals; ARRIVE guidelines recommended for reporting animal research

Laboratory animals	Drosophila melanogaster not classified as animals. Ethics approval not required. Animals from stock centers.
Wild animals	n/a
Field-collected samples	n/a
Ethics oversight	n/a

Note that full information on the approval of the study protocol must also be provided in the manuscript.
