# Supplementary material for: Drosophila melanogaster behaviour changes in different social environments based on group size and density
Source: Commun Biol. 2020 Jun 12;3:304. doi: 10.1038/s42003-020-1024-z (PMC7293324; doi:10.1038/s42003-020-1024-z)
Supplement: Supplementary file 4 — Description of Additional Supplementary Files [file 42003_2020_1024_MOESM4_ESM.pdf]

Description of Additional Supplementary Files

File Name: **Supplementary Data 1**
Description: Trial Data for Social Behaviour Measurements and Interaction Criteria
